# Mechanical and thermal properties of bacterial-cellulose-fibre-reinforced Mater-Bi^®^ bionanocomposite

**DOI:** 10.3762/bjnano.4.37

**Published:** 2013-05-23

**Authors:** Hamonangan Nainggolan, Saharman Gea, Emiliano Bilotti, Ton Peijs, Sabar D Hutagalung

**Affiliations:** 1 Laboratory of Physical Chemistry, Department of Chemistry, Faculty of Mathematics and Natural Sciences, University of Sumatera Utara (USU), Medan 20155, Indonesia; 2 School of Engineering and Material Sciences, Queen Mary University of London, Mile End Road, London E1 4NS, United Kingdom; 3 School of Materials and Mineral Resources Engineering, Universiti Sains Malaysia, 14300 Nibong Tebal, Penang, Malaysia

**Keywords:** bacterial cellulose, bio-nanocomposites, Mater-Bi, mechanical, thermal

## Abstract

The effects of the addition of fibres of bacterial cellulose (FBC) to commercial starch of Mater-Bi^®^ have been investigated. FBC produced by cultivating *Acetobacter xylinum* for 21 days in glucose-based medium were purified by sodium hydroxide 2.5 wt % and sodium hypochlorite 2.5 wt % overnight, consecutively. To obtain water-free BC nanofibres, the pellicles were freeze dried at a pressure of 130 mbar at a cooling rate of 10 °C min^−1^. Both Mater-Bi and FBC were blended by using a mini twin-screw extruder at 160 °C for 10 min at a rotor speed of 50 rpm. Tensile tests were performed according to ASTM D638 to measure the Young’s modulus, tensile strength and elongation at break. A field emission scanning electron microscope was used to observe the morphology at an accelerating voltage of 10 kV. The crystallinity (*T*_c_) and melting temperature (*T*_m_) were measured by DSC. Results showed a significant improvement in mechanical and thermal properties in accordance with the addition of FBC into Mater-Bi. FBC is easily incorporated in Mater-Bi matrix and produces homogeneous Mater-Bi/FBC composite. The crystallinity of the Mater-Bi/FBC composites decrease in relation to the increase in the volume fraction of FBC.

## Introduction

Farm products offer a wide range of commodities such as cellulose, starch, rubber, and other materials that are environmentally friendly. Starch, one of the promising natural materials, most abundantly available after cellulose, has been considered a good candidate in thermoplastic technology [1]. The low price and the availability of starch in addition to its very favourable environmental profile, have, in the past 15 years, aroused a renewed interest in starch-based polymers as an attractive alternative to polymers based on petrochemicals [2].

Mater-Bi^®^ is derived from renewable raw materials of agricultural origin and non-genetically modified starch. Mater-Bi is biodegradable and compostable in soil, and fresh and salt water. Mater-Bi is a commercially available thermoplastic starch/polyethylene-vinyl alcohol (PEVOH) [3] produced by Novamont, a leading European company and pioneer in the field of bioplastics in Italy with a capacity of 60,000 tons per annum. Various kinds of products made from Mater-Bi have been marketed in accordance with the specifications of its use due to the fact that it is derived from a natural raw material and, so, has a low impact on the environment.

Composites of Mater-Bi with biodegradable fibres, particularly plant cellulose, have been developed. The use of flax cellulose pulp with Mater-Bi produces better mechanical properties and higher thermal stability [4]. Short fibres of sisal added in the range from 5 to 20% have been able to raise the resistance of the composite to the initiation of cracks and progression of fractures compared to its pure matrix [5], while its biodegradability is shown by the fact that being buried in soil for just a month has the effect of reducing its mechanical properties as a result of damage to the fibre/matrix interface [6].

Cellulose is the main component of plant cell walls. Some bacteria produce cellulose (celled biocellulose or bacterial cellulose). Plant cellulose and bacterial cellulose (BC) have the same chemical structure, but different physical and chemical properties. BC is produced by culturing a strain of *Acetobacter xylinum*, reclassified as the genus *Gluconacetobacter,* which is typically found on decaying fruits, vegetables, vinegar, fruit juices, and alcoholic beverages. The bacteria of this family convert ethanol to acetic acid. BC has recently received extensive attention from researchers due to its unique properties, such as high water capacity, high crystallinity, ultrafine fibre networks with a diameter of 20–100 nm, high purity (which is distinguished from plant cellulose), and high tensile strength [7–10]. The isotropic Young’s modulus of a BC sheet is about 20 GPa [11–13]. Meanwhile, the modulus of a single BC fibre estimated by Raman spectroscopy techniques is 130 GPa [14–15]. Therefore BC has been used extensively in many fields including wound dressings, optical displays, implants, drug delivery and tissue engineering.

Until now there have been no reports of bionanocomposites of Mater-Bi reinforced by the fibre of bacterial cellulose (FBC), which is the main goal of this work. It is expected that the composite of BC and Mater-Bi will produce better mechanical properties and higher thermal stability. Therefore, composites base on Mater-Bi NF01U, a product from Novamont, with BC were prepared and their mechanical, thermal and morphological properties tested.

## Results and Discussion

**Figure 1:**
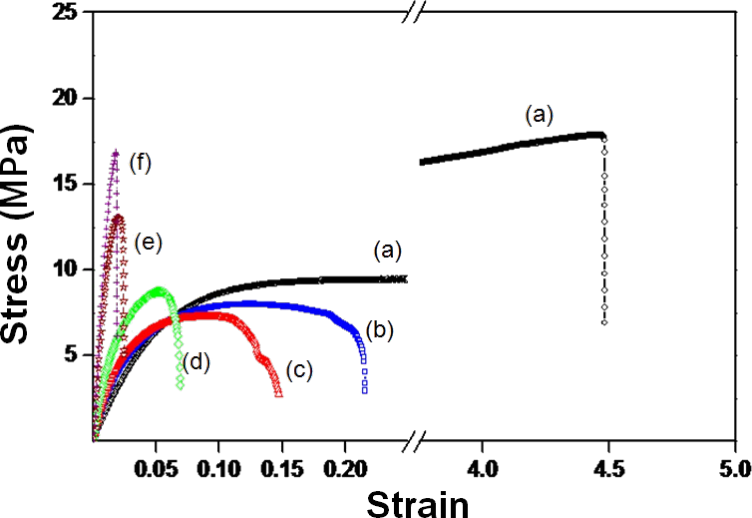
Stress–strain curves of Mater-Bi and Mater-Bi/FBC bionanocomposites: (a) pure Mater-Bi, (b) Mater-Bi/FBC (90:10), (c) Mater-Bi/FBC (80:20), (d) Mater-Bi/FBC (70:30), (e) Mater-Bi/FBC (60:40), and (f) Mater-Bi/FBC (50:50).

The mechanical properties of Mater-Bi as well as bionanocomposites of Mater-Bi/FBC, including the Young’s modulus, tensile strength, and elongation at break are shown in Figure 1*.* By adding 10% of FBC into the matrix of Mater-Bi the Young’s modulus increases by up to 200% compared to Mater-Bi, indicating the effectiveness of the reinforcement. The greater the volume fraction of FBC, the greater the stiffness of the composites. The improvement in mechanical properties is due to the quantitative increase in H-bonds that comes from BC, and is accompanied by an improvement in the fibre–matrix interaction [16]. The addition of FBC to the Mater-Bi matrix gives a significant change in brittleness with a strain at break of 21% at 10% BC. Moreover, pull-out of the BC fibres contributes to the composite toughness.

**Figure 2:**
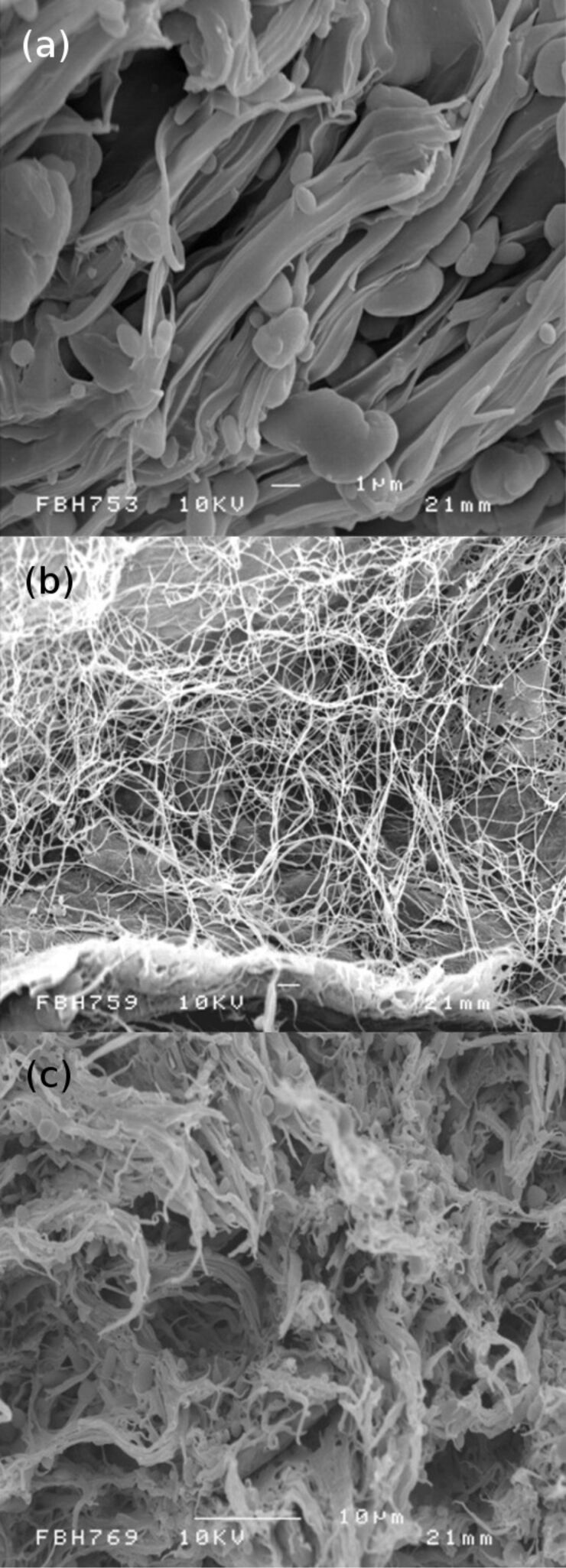
SEM images of (a) pure Mater-Bi, (b) FBC and (c) Mater-Bi/FBC.

The addition of FBC into Mater-Bi containing PEVOH as plasticizer for the preparation of Mater-Bi/FBC bionanocomposites showed morphological changes on the surface, as shown in Figure 2*.* It can be seen that FBC is easily incorporated in the Mater-Bi matrix and gives a good dispersion (Figure 2(c)). The surface of Mater-Bi/FBC in Figure 2(c) seems to be smoother than that of pure Mater-Bi (Figure 2(a)) and also shows that FBCs (Figure 2(b)) were blended and dispersed uniformly into Mater-Bi as its matrix. The network of fibres found in FBC leads to an increase in toughness through energy absorption processes such as fibre pull-out. This increased in toughness is confirmed by the higher elongation at break of Mater-Bi/FBC in accordance with the increased of the volume fraction of FBC.

**Figure 3:**
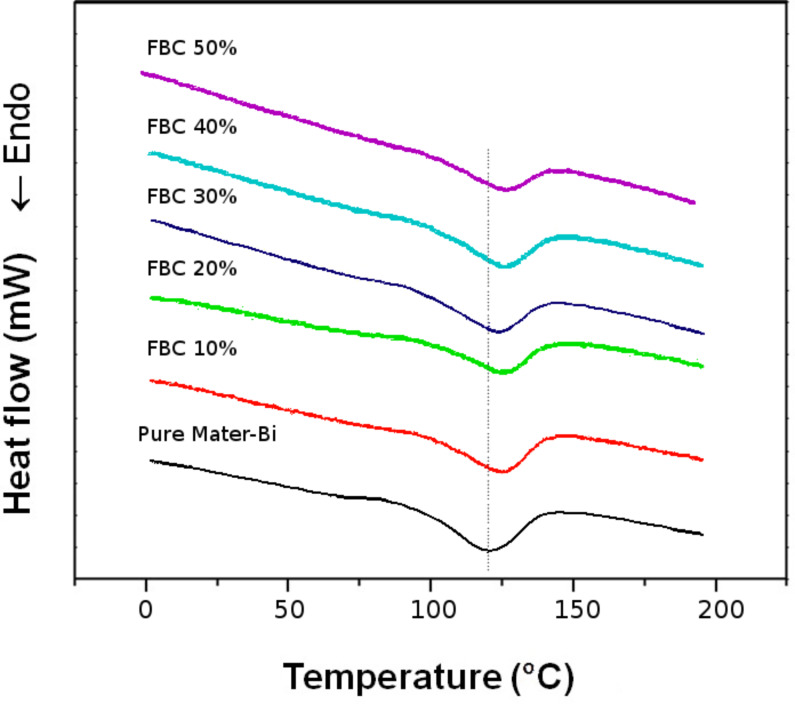
DSC Thermograms of the second crystallinity and melting of Mater-Bi and Mater-Bi/FBC.

The DSC thermograms of thermal behaviour including crystallinity and melting point of pure Mater-Bi and Mater-Bi/FBC composites are shown in Figure 3, and the results are listed in Table 1. The crystallinity of Mater-Bi decreases when blended with FBC as a reinforcing agent, perhaps as a result of the increased hydrophobicity of the composites due to the introduction of hydrophobic groups by hydroxypropylation [17]. Meanwhile, the melting point of Mater-Bi/FBC composites increase when more FBC was added as a reinforcing agent (Table 1). The same phenomena is observed when short pulp fibre is incorporated into the corn starch with glycerol used as a plasticizer [17], and this is closely related to the decrease in crystallinity of the composite with the increase in FBC.

**Table 1:** The thermal properties of Mater-Bi and Mater-Bi/FBC bionanocomposites.

Mater-Bi/FBC [%]	Crystallinity [%]	Melting Point [°C]

100/0	4.25	120
90/10	3.31	124
80/20	2.53	125
70/30	1.92	125
60/40	1.46	126
50/50	0.83	127

## Conclusion

The morphology of Mater-Bi is changed by adding FBC as a reinforcement material. FBC is easily incorporated into a Mater-Bi matrix and gives a well dispersed Mater-Bi/FBC composite. The mechanical properties of Mater-Bi are improved by adding FBC. The stiffness of the composite increased significantly with the volume fraction of FBC. The enhancement of the properties of Mater-Bi/FBC bionanocomposites is caused by the improvement of the fibre–matrix interface as shown in the SEM images. DSC thermogram shown that the crystallinity of the Mater-Bi/FBC composites decrease in relation to the increase in the volume fraction of FBC.

## Experimental

The strains of *Acetobacter xylinum* used for the production of BC were supplied by the Microbiology Laboratory of the Institute of Agriculture, Bogor, Indonesia. The chemicals used for the culture medium were glucose, ammonium sulfate, potassium hydrogen orthophosphate, magnesium sulfate and yeast extract. In the purification process, sodium hydroxide, sodium hypochlorite and the chemicals mentioned above were used as received from VWR. Additional vitamins such as B_1_, B_3_, and B_5_ were used to assist growth [11]. Both a smooth, nonwoven cloth and a wire 200 mesh sieve were used to avoid slippage of the BC gel when a load was applied. Mater-Bi grade NF01U kindly supplied by Novamont SpA, Italy was also investigated.

The culture medium was prepared according to the method described previously [11]. For every litre of distilled water, 50 g glucose, 5 g ammonium sulfate, 4 g potassium hydrogen orthophosphate, 5 g yeast extract, and 0.1 g magnesium sulfate were added. This culture medium was autoclaved at 121 °C for 2 h, and once cooled to ambient temperature, vitamins B_1_, B_2_, and B_5_ were added. The acidity of the medium was adjusted to pH = 4 by using glacial acetic acid. The medium was inoculated by sowing the bacteria with a handle-loop on the surface of the agar and putting it into the bottle. In order to ensure a good distribution of the bacteria and to remove the remaining agar from the bacteria, the bottle was shaken for 4 h, before being placed in an incubator at 28 °C for 21 days in a quiet room. After harvesting, the pellicles were cleaned by a two-step treatment by immersing overnight in NaOH 2.5 wt % and then continuing for another night in NaOCl 2.5 wt % [18].

Freeze-dried BC used in this work was reported previously [12]. Mater-Bi was used as received without any further treatment. Mater-Bi was blended with FBC to prepare a Mater-Bi/FBC bionanocomposite by using a mini twin-screw extruder at 160 °C for 10 min at the rotor speed of 50 rpm with a volume fraction of FBC of 0, 10, 20, 30, 40, and 50%. For the preparation of test specimens, the compounds above were compression moulded into dumbbell-shaped tensile bars by using a hot-press, at 120 °C and 55 kN for 5 min. The dimensions of the sample were a thickness of 1.25 mm and width of 2.95 mm and a gauge length of 30 mm, following the ASTM D638 method.

Tensile tests were performed by using an Instron tensile testing machine according to ASTM D638 equipped with a 1 kN static load cell. The tests were used to measure Young’s modulus, tensile strength and elongation at break. At least five replicate samples were tested for each composition. The width and the thickness of each sample were measured before testing and the approximate values were 3.0 mm and 1.25 mm, respectively. A test speed of 1 mm min^−1^ was used throughout.

A JEOL JSM-6300F field emission scanning electron microscope (FE-SEM) was used to observe the morphology of the FBC/Mater-Bi composites. All samples were coated with gold and observed by using an accelerating voltage of 10 kV.

Differential scanning calorimetric (DSC) experiments were carried out by using a Perkin Elmer Pyris DSC-7 calorimeter. The average sample weights of approximately 15.0–17.5 mg were placed in the aluminium crucible. Heating rates of 20 °C min^−1^ were used in the temperature range 20–400 °C. The crystallinity (*T*_c_) and melting temperature (*T*_m_) were recorded as the inflection point of the increment of specific heat and as the peak value of the endothermic process in the DSC curve, respectively [19].
